# The use of human donor milk

**DOI:** 10.1136/bmj.m4243

**Published:** 2020-11-17

**Authors:** Hoang Thi Tran, Tuan T Nguyen, Roger Mathisen

**Affiliations:** 1 Da Nang Hospital for Women and Children, Da Nang, Vietnam; 2 Alive & Thrive Southeast Asia, FHI 360, Hanoi, Vietnam

**What you need to know**

- When a mother’s own milk is unavailable for preterm or low birthweight infants, pasteurised donor milk protects against necrotising enterocolitis compared with formula milk
- Pasteurised donor milk can also be used to supplement the mother’s own milk in order to meet an infant’s nutritional needs and can be administered in the same ways as the mother’s own expressed milk
- The only absolute contraindication to the use of pasteurised donor milk is galactosaemia. Mothers with active pulmonary tuberculosis or herpes simplex lesions on the breast should be encouraged to feed expressed breast milk, while mothers with covid-19 should be advised to continue breastfeeding

*A 25 year old woman gave birth to her first child prematurely, at 27 weeks gestation, at Da Nang Hospital for Women and Children, in central Vietnam. The male infant weighed 980 grams and required continuous positive airway pressure respiratory support in the neonatal intensive care unit. Neonatal care nurses provided breastfeeding counselling and support to the mother, and the infant received mother’s own milk as well as pasteurised donor milk in the first four days of life. From the fifth day of life, the mother was able to produce enough breast milk to meet her son’s nutritional needs. During the hospital stay, the neonatal nurses encouraged the mother to breastfeed, store breast milk for her son, and donate her surplus breast milk to the human milk bank. She became a breast milk donor when her son was 45 days old and still hospitalised. The mother continued donating breast milk once she returned home, ultimately donating 62 L of breast milk over five months. The mother was proud to be a breast milk donor to newborns in need.*

In line with global recommendations from the World Health Organization (WHO), the UK National Institute for Health and Care Excellence (NICE) and other national infant feeding guidelines emphasise that breast milk is the best nutrition for infants, and infants should be exclusively breastfed in the first 6 months of life.^1^ ^2^ Indeed, high quality trials and meta-analyses show that breastfeeding is associated with reduced child mortality from diarrhoea and pneumonia and reduced risk for childhood obesity and diabetes,^3^ as well as economic savings.^4^ However, some infants do not have access to their biological mother’s milk for a range of reasons. Historically, wet nursing and animal milks were used to feed these infants.^5^ Innovation in infant bottles, advances in hygiene, and the invention of formula milks over the 19th century greatly expanded artificial infant feeding, and breastfeeding rates declined globally in response to greater availability and marketing of infant formula milk.^5^ Since 1979, however, WHO and Unicef have recommended the use of pasteurised human milk from a human milk bank to feed low birthweight and preterm infants as the first alternative when mothers are unable to provide their own milk.^6^

## What is the rationale for using human donor milk?

Pasteurised donor milk provides the nutritional and immunologic benefits of breast milk and reduces infectious complications in preterm or low birthweight infants compared with formula milk. In a systematic review, the use of pasteurised donor milk was associated with a 46% reduction in the incidence of necrotising enterocolitis compared with infant formula (term or preterm) in preterm or low birthweight infants.^7^ Pasteurised human breast milk has also been associated with a 19% reduction in the odds of developing sepsis for every 10 mL/kg consumed each day in the first 28 days of life in very low birthweight infants compared with infant formula.^8^ Among preterm infants who receive some of their mother’s own milk, those who were supplemented with donor human milk had a 22% lower incidence of bronchopulmonary dysplasia and required almost three fewer days of ventilator support than preterm infants who were supplemented with preterm formula.^9^

The use of donor breast milk and presence of a human milk bank are also associated with higher rates of breastfeeding beyond delivery, even for infants who are admitted to neonatal intensive care units.^9^ ^10^ In a study of 83 neonatal intensive care units in Italy, for example, the prevalence of exclusive breastfeeding at discharge was 14 percentage points higher in the units with a human milk bank than in those without.^11^ And, in the US, a small study of 122 infants hospitalised for the treatment of hypoglycaemia, hyperbilirubinemia, and >8% weight loss at 40 hours of life found that newborns who received donor breast milk had five times greater odds of exclusive breastfeeding at 6 months old compared with those who received formula.^12^

## What is a human milk bank?

A human milk bank is a service that recruits breast milk donors; collects, pasteurises, and stores donor milk; tests the milk for bacterial contamination; and then distributes donor milk to recipient infants and families.^13^ Human milk donors are typically healthy breastfeeding postpartum women with a surplus of milk who volunteer to donate milk.^13^ Pasteurisation essentially eliminates the risk of toxic, bacterial, or viral (including HIV and SARS-CoV-2) contamination while maintaining the nutritious and immunologic qualities of breast milk.^13^ ^14^ ^15^

The global human milk bank network expanded from less than 10 in 1979 to 700 human milk banks in more than 60 countries in 2020.^13^ ^16^ The demand for donor milk from even new milk banks can be high. For example, in its first two years of operation, the first human milk bank in Vietnam at Da Nang Hospital for Women and Children—a facility with about 15 000 births each year—collected 4400 L of milk from 315 donors and distributed more than 3000 L of pasteurised human milk to 8100 babies.^17^ In Brazil in 2019, a network of 224 human milk banks collected 223 000 L of milk from 189 000 donors and distributed 192 000 L of pasteurised human milk to 215 000 babies.^18^

## When should pasteurised donor milk be recommended?

If a mother’s own milk is not available, breastfeeding is contraindicated, or an infant’s mother is unable to provide sufficient quantities of her own breast milk after being provided with lactation support, pasteurised donor milk is indicated as a first alternative for any infant (box 1).^2^ ^13^ ^19^ ^25^ Infants with severe medical conditions, premature infants, or very low birthweight babies (<1500 g) are prioritised when demand for donor milk exceeds supply.^2^ ^13^ ^19^ Parental consent is typically required before the use of donor milk in most human milk banks, including those from Brazil, France, India, and North America.^13^

Box 1 **Common indications for use of pasteurised donor milk^13^ ^19^**

- Mother is absent due to separation, sickness, death, or abandonment^13^ ^19^
- Mothers infected with HIV (in specific contexts), human T cell lymphotropic virus type I or II, untreated brucellosis, Ebola virus disease, or breast cancer with double mastectomy^20^ ^21^
- Mothers who require medications that are contraindicated in breastfeeding such as amiodarone, chemotherapeutic or antineoplastic agents, chloramphenicol, ergotamine, gold salts, phenindione, radioactive pharmaceuticals, retinoids, tetracyclines (use for more than 3 weeks), and certain psychotropic medications^22^ ^23^ ^24^
- Mothers who use phencyclidine, cocaine, and cannabis may be candidates out of concern for long term neurobehavioral development in the infant^22^ ^23^ ^24^
- Lesbian, gay, bisexual, and transgender (LGBT) couples, adoptive or other parents without lactating capacity^13^ ^19^
- Mothers who do not have enough breast milk during the first days after birth despite lactation support^13^ ^19^

## Are there any contraindications to pasteurised donor milk?

The only absolute contraindication to the use of pasteurised donor milk is classic galactosaemia, a rare congenital disorder of galactose metabolism (the sugar is found in breast milk and in normal formula).^20^ Expressed mother’s own milk is recommended for infants born to mothers with active, untreated pulmonary tuberculosis diagnosed before or after delivery, herpes simplex lesions on the breast, varicella from five days before through to two days after delivery, or H1N1 influenza.^22^ ^26^ Both WHO and the US Centres for Disease Control and Prevention recommended that mothers infected with SARS-CoV-2 should continue breastfeeding while using infection control practices such as handwashing and wearing a face mask to limit the risk of infecting their infant.^27^ ^28^

### Is pasteurised donor milk recommended for infants of women infected with HIV?

Countries typically use or adapt guidelines on HIV infection and infant feeding from WHO,^21^ the American Academy of Paediatrics,^22^ and other organisations such as the British HIV Association.^29^ In high income countries such as the US and UK, mothers living with HIV regardless of maternal viral load and antiretroviral therapy are recommended not to breastfeed their children. Thus, pasteurised donor milk or infant formula is recommended.^22^ ^29^ In other settings, where clean water supply is insecure or human milk banks are not operational, WHO and Unicef recommend that women known to be infected with HIV and whose infants are not infected or of unknown HIV status exclusively breastfeed their infants for the first 6 months of life, introducing appropriate complementary foods thereafter, and continue to breastfeed for at least 12 months.^21^ We recommend that clinicians engage HIV-positive women in early discussions of risks and benefits of various infant feeding modalities in line with local guidelines.

## How to feed pasteurised donor milk to infants

Pasteurised donor milk needs to be defrosted before use (box 2). Like any expressed breast milk, pasteurised donor milk can be fed by health staff or family members by means of cup, spoon, via small tubes that the infant suckles along with a nipple, or via gastric tubes, and should be given according to clinical guidelines.^13^ The required volume of donor milk is based on the newborn’s age, gestational age, and weight, as well as tolerance of feeding and weight gain (such as 15-20 g/kg/day) and represents the difference between recommended fluid intake and the available quantity of mother’s own milk (and transfused parenteral nutrition, if applicable). The recommended amount of fluid intake (table 1)^30^ is distributed across the number of feeds each day (such as 1-3 hourly feeds).^25^ ^30^ Decisions about the need for ongoing human donor milk are regularly reviewed, taking into account infant growth, fluid balance, and nutritional requirements.^25^ ^30^

Box 2 **How to prepare frozen pasteurised donor milk for feeding^1^ ^13^ ^25^**

- Defrost donor milk containers slowly over 24 hours in a refrigerator at 4°C (with care to prevent the milk from reaching 8°C to prevent growth of microorganisms)
- For urgent, exceptional cases, containers can be defrosted at room temperature or (less preferable) under warm water
- Milk defrosted in the refrigerator should be used within 24 hours
- Milk that underwent quick defrosting should be used right away and should not be re-refrigerated

**Table 1:** Recommended fluid intake (mL/kg/day) during the first 30 days of life in preterm, low birthweight neonates^30^

Preterm neonate birth weight	Phase I (transition)					Phase II (intermediate) Days 6-10	Phase III (stable growth) Days 11-30
	Day 1	Day 2	Day 3	Day 4	Day 5		
>1500 g	60-80	80-100	100-120	120-140	140-160	140-160	140-160
1000-1500 g	70-90	90-110	110-130	130-150	160-180	140-160	140-160
<1000 g	80-100	100-120	120-140	140-160	160-180	140-160	140-160

Encourage mothers of infants in a neonatal intensive care unit to express breast milk as soon as they can.^19^ Once infants can coordinate sucking, swallowing, and breathing (around 32-34 gestational weeks), mothers can trial direct breastfeeding.^31^ In cases where mothers are unable to breastfeed, pasteurised donor milk can be provided beyond hospital discharge.

### Use of donor milk complements other strategies to support breastfeeding

Pasteurised donor milk can and should be provided simultaneously with other measures to promote breastfeeding and newborn health in the immediate postpartum period, including skin-to-skin for healthy newborns and “kangaroo mother care” (prolonged skin-to-skin contact provided by mother or family members) for low birthweight or preterm infants in a stable condition.^32^ Although evidence supports the use of pasteurised donor milk in high and low income settings, and human milk banks are growing in number, the major limitations are the operating cost and, in some settings, societal norms relating to milk sharing.^17^ ^33^

**Human donor milk recipient story**

A 32 year old mother gave birth vaginally to her third child at 32 weeks gestation, weighing 1800 g, in a provincial hospital in the Central Highlands of Vietnam. He was diagnosed with respiratory distress and received continuous positive airway pressure (CPAP) for 15 days. Antibiotics and prolonged parenteral fluid were given with no enteral feeding. The mother was separated from her son except for some daily visits, as per routine practice in that hospital for babies requiring respiratory support. On day of life 20, he was given some millilitres of his mother’s milk, but did not tolerate this feed.

He was transferred to Da Nang Hospital for Women and Children at 23 days of life to undergo evaluation for suspected intestinal obstruction. Once intestinal obstruction was ruled out, the baby was transitioned from parenteral nutrition to gradually increasing volumes of pasteurised donor milk through nasogastric tubes as his mother did not have breast milk. The mother was supported to provide kangaroo mother care, and she also received psychological and lactation support. She was able to re-initiate milk production, and her baby breastfed directly at 30 days of life. He was discharged home at 40 days of life to continue breastfeeding with pasteurised donor milk supplementation.

His mother continued breastfeeding and, by 65 days of life, was able to meet the full nutritional needs of her son with her own breast milk. She felt happy that she was able to breastfeed her son and saved about 2.5 million Vietnamese Dong (~US$110) per month, which is more than half of the parent’s income, by avoiding the use of infant formula.

**How this article was made**

This article was created using a combination of guidelines and expert advice (both neonatologists and general practitioners) and established literature (through Medline and Google Scholar literature searches up to August 2020 on “human milk bank, breastfeeding, preterm, and low birth weight.”

**Education into practice**

- Are you familiar with your nearest human milk bank?
- Can you think of infants in your practice who might have benefitted from pasteurised donor milk?
- How could health staff maximise the benefits of a human milk bank?

**How patients were involved in the creation of this article**

The patient stories, from one donor and one recipient, were selected from a pool of case studies routinely collected by the Human Milk Bank in Da Nang Hospital for Women and Children. These stories show the importance of pasteurised donor milk for sick and small babies, helping mothers to feed their babies exclusively with human breast milk and maintain lactation during hospital stay, at discharge, and beyond.

## Notes

Provenance and peer review: Commissioned; externally peer reviewed.

Dissemination: The information will be disseminated to ministries of health, medical schools, professional medical associations, major maternity and paediatric hospitals, and other Alive and Thrive partners in South and South-east Asia and East and West Africa. We will also disseminate the information within global and regional networks focusing on human milk bank services and neonatal health.

## References

[1] Centre for Clinical Practice at NICE. Donor breast milk banks: The operation of donor milk bank services. 2010. https://www.nice.org.uk/guidance/cg93/evidence/full-guideline-243964189. 22319806

[2] WHO Guidelines on optimal feeding of low birth-weight infants in low-and middle-income countries. World Health Organization, 2011. 26042325

[3] Victora CG Bahl R Barros AJ Lancet Breastfeeding Series Group Breastfeeding in the 21st century: epidemiology, mechanisms, and lifelong effect. Lancet 2016;387:475-90. 10.1016/S0140-6736(15)01024-7 26869575

[4] Walters DD Phan LTH Mathisen R The cost of not breastfeeding: global results from a new tool. Health Policy Plan 2019;34:407-17. 10.1093/heapol/czz050 31236559 PMC6735804

[5] Stevens EE Patrick TE Pickler R A history of infant feeding. J Perinat Educ 2009;18:32-9. 10.1624/105812409X426314 20190854 PMC2684040

[6] WHO WHO/UNICEF meeting on infant and young child feeding. J Nurse Midwifery 1980;25:31-9. 10.1016/0091-2182(80)90051-8 6900060

[7] Quigley M Embleton ND McGuire W Formula versus donor breast milk for feeding preterm or low birth weight infants. Cochrane Database Syst Rev 2018;6:CD002971. 10.1002/14651858.CD002971.pub4 29926476 PMC6513381

[8] Patel AL Johnson TJ Engstrom JL Impact of early human milk on sepsis and health-care costs in very low birth weight infants. J Perinatol 2013;33:514-9. 10.1038/jp.2013.2 23370606 PMC3644388

[9] Villamor-Martínez E Pierro M Cavallaro G Mosca F Kramer BW Villamor E Donor human milk protects against bronchopulmonary dysplasia: a systematic review and meta-analysis. Nutrients 2018;10:E238. 10.3390/nu10020238 29461479 PMC5852814

[10] Williams T Nair H Simpson J Embleton N Use of donor human milk and maternal breastfeeding rates: a systematic review. J Hum Lact 2016;32:212-20. 10.1177/0890334416632203 26887844

[11] Arslanoglu S Moro GE Bellù R Presence of human milk bank is associated with elevated rate of exclusive breastfeeding in VLBW infants. J Perinat Med 2013;41:129-31. 10.1515/jpm-2012-0196 23241582

[12] Merjaneh N Williams P Inman S The impact on the exclusive breastfeeding rate at 6 months of life of introducing supplementary donor milk into the level 1 newborn nursery. J Perinatol 2020;40:1109-14. 10.1038/s41372-020-0657-6 32231257

[13] PATH Strengthening human milk banking: a resource toolkit for establishing and integrating human milk bank programs--A Global Implementation Framework. PATH, 2019.

[14] Hartmann BT Ensuring safety in donor human milk banking in neonatal intensive care. Clin Perinatol 2017;44:131-49. 10.1016/j.clp.2016.11.006 28159201

[15] Shenker N Virtual Collaborative Network of Human Milk Banks and Associations Maintaining safety and service provision in human milk banking: a call to action in response to the COVID-19 pandemic. Lancet Child Adolesc Health 2020;4:484-5. 10.1016/S2352-4642(20)30134-6 32573440 PMC7202855

[16] Tableau Public. Human milk bank global map. 2020. https://public.tableau.com/profile/human.milk.bank.global.map#!/vizhome/HumanMilkBankGlobalMap_0/HumanMilkBankGlobalMap.

[17] Mansen K Nguyen TT Nguyen NQ Strengthening newborn nutrition through establishment of the first human milk bank in Vietnam. J Hum Lact 2020; 10.1177/0890334420948448. 32833551 PMC7907997

[18] Oswaldo Cruz Foundation Brazilian Network of Human Milk Banks documents series monitoring agenda 2030 - rBLH in data. Brazilian Network of Human Milk Banks, 2020.

[19] Brandstetter S Mansen K DeMarchis A Nguyen Quyhn N Engmann C Israel-Ballard K A decision tree for donor human milk: an example tool to protect, promote, and support breastfeeding. Front Pediatr 2018;6:324. 10.3389/fped.2018.00324 30430103 PMC6220111

[20] Lawrence RM Circumstances when breastfeeding is contraindicated. Pediatr Clin North Am 2013;60:295-318. 10.1016/j.pcl.2012.09.012 23178071

[21] World Health Organization. Updates on HIV and infant feeding: guideline. WHO, Unicef, 2016. https://www.who.int/maternal_child_adolescent/documents/hiv-infant-feeding-2016/en/. 27583316

[22] Eidelman AI Breastfeeding and the use of human milk: an analysis of the American Academy of Pediatrics 2012 Breastfeeding Policy Statement. Breastfeed Med 2012;7:323-4. 10.1089/bfm.2012.0067 22946888

[23] National Library of Medicine. Drugs and Lactation Database (LactMed). 2006. https://www.ncbi.nlm.nih.gov/books/NBK501922/.

[24] Sachs HC Committee on Drugs The transfer of drugs and therapeutics into human breast milk: an update on selected topics. Pediatrics 2013;132:e796-809. 10.1542/peds.2013-1985 23979084

[25] Granger C, Powls A, Simpson J, Cairns L. Enteral feeding of preterm infants. NHS Greater Glasgow and Clyde. 2017. https://www.clinicalguidelines.scot.nhs.uk/ggc-paediatric-guidelines/ggc-guidelines/neonatology/enteral-feeding-of-preterm-infants/.

[26] Mittal H Das S Faridi MM Management of newborn infant born to mother suffering from tuberculosis: current recommendations & gaps in knowledge. Indian J Med Res 2014;140:32-9. 25222775 PMC4181157

[27] Centers for Disease Control and Prevention. Breastfeeding and special circumstances. 2020. https://www.cdc.gov/breastfeeding/breastfeeding-special-circumstances/index.html.

[28] World Health Organization. Breastfeeding and COVID-19. 2020. https://www.who.int/news-room/commentaries/detail/breastfeeding-and-covid-19.

[29] Gilleece DY Tariq DS Bamford DA British HIV Association guidelines for the management of HIV in pregnancy and postpartum 2018. HIV Med 2019;20(Suppl 3):s2-85. 10.1111/hiv.12720 30869192

[30] Jochum F Moltu SJ Senterre T Nomayo A Goulet O Iacobelli S ESPGHAN/ESPEN/ESPR/CSPEN Working Group on Pediatric Parenteral Nutrition ESPGHAN/ESPEN/ESPR/CSPEN guidelines on pediatric parenteral nutrition: fluid and electrolytes. Clin Nutr 2018;37(6 Pt B):2344-53. 10.1016/j.clnu.2018.06.948 30064846

[31] Browne JV Ross ES Eating as a neurodevelopmental process for high-risk newborns. Clin Perinatol 2011;38:731-43. 10.1016/j.clp.2011.08.004 22107901

[32] World Health Organization Kangaroo mother care: a practical guide. WHO, 2003.

[33] Alnakshabandi K Fiester A Creating religiously compliant milk banks in the Muslim world: a commentary. Paediatr Int Child Health 2016;36:4-6. 10.1080/20469047.2015.1110336 26750779

